# Ionic Channels as Targets for Drug Design: A Review on Computational Methods

**DOI:** 10.3390/pharmaceutics3040932

**Published:** 2011-12-09

**Authors:** Gregorio Fernández-Ballester, Asia Fernández-Carvajal, José Manuel González-Ros, Antonio Ferrer-Montiel

**Affiliations:** Instituto de Biología Molecular y Celular, Universidad Miguel Hernández, Alicante 03202, Spain

**Keywords:** virtual screening; ion channel; channelopathies; quantitative structure, activity relationships; homology models; docking; pharmacology; *in silico*, *in vitro*, drug discovery, computational approaches

## Abstract

Ion channels are involved in a broad range of physiological and pathological processes. The implications of ion channels in a variety of diseases, including diabetes, epilepsy, hypertension, cancer and even chronic pain, have signaled them as pivotal drug targets. Thus far, drugs targeting ion channels were developed without detailed knowledge of the molecular interactions between the lead compounds and the target channels. In recent years, however, the emergence of high-resolution structures for a plethora of ion channels paves the way for computer-assisted drug design. Currently, available functional and structural data provide an attractive platform to generate models that combine substrate-based and protein-based approaches. *In silico* approaches include homology modeling, quantitative structure-activity relationships, virtual ligand screening, similarity and pharmacophore searching, data mining, and data analysis tools. These strategies have been frequently used in the discovery and optimization of novel molecules with enhanced affinity and specificity for the selected therapeutic targets. In this review we summarize recent applications of *in silico* methods that are being used for the development of ion channel drugs.

## 1. Introduction

Ion channels are proteins that allow the passage of charged ions through hydrophobic membranes. These proteins are sophisticated devices that conduct ions with exquisite specificity at speeds close to the limit of diffusion, under very tight regulation.

Because of the contribution of ion channels to the pathophysiology of several human diseases, these membrane proteins are the targets of diverse drugs, from antiepileptics to analgesics, although they still remain relatively underexploited for therapeutic interventions. Furthermore, the overexpression of ionic channels in some cancer cells prompted a renewed interest in these proteins as targets of novel anticancer drugs. Therapeutically important channels include voltage-gated ion channels for potassium, sodium, chloride and calcium that are present in the outer membrane of a variety of cells such as those responsible for the electrical excitability and signaling in nerve and muscle cells [1]. In addition, these membrane proteins are also involved in drug absorption, distribution, metabolism, excretion, and toxicity (ADME/Tox), thus defining several important aspects of pharmacodynamics (PD) and pharmacokinetics (PK) [2], and contributing frequently to the unwanted side effects of drugs.

At a molecular level, most of the channels are poorly characterized because of the difficulties associated with the recombinant expression, purification and crystallization of membrane proteins [3]. Moreover, the discovery of lead compounds or preclinical candidates from sophisticated experimental High Throughput Screening (HTS) approaches did not result in the promised outcome [4]. *In silico* approaches are useful tools to override such difficulties because they readily generate structure-function relationships facilitating the understanding of channel function in terms of their underlying protein structure. The term ‘virtual screening’ (VS) was coined in an attempt to show the computer approaches as an alternative to HTS, where the compounds (the ligands) are predicted to bind to the target (the receptor). The list of advantages of VS over HTS includes the low cost, the overriding of the limited solubility and/or aggregation, or even the existence of the known compounds [4]. However, VS as a knowledge-driven approach [4] requires three-dimensional information (3D structures) given by crystallographic data, nuclear magnetic resonance (NMR), homology modeling or, at least, ligand references with available bioactive information [5].

Over the past years significant computational (*in silico*) methods have been developed and widely applied to drug development on several proteins [6], including ion channels. Targeted proteins differ widely in architecture and function, ranging from highly selective ion channels (Kv1.2) to less selective channels, as nicotinic acetylcholine receptors (nAChR) or acid sensing ion channels (ASIC1), and to channels, such as SecY, that conduct entire proteins [7–10]. A recent review has discussed the various Quantitative Structure Activity Relationship (QSAR) methods such as pharmacophores, Comparative Molecular Field Analysis (CoMFA), Supported Vector Machine (SVM), 2D-QSAR, Genetic Programming, Self-Organizing Maps and recursive partitioning, that have been applied to most ion channels in the absence of crystal structures [11]. To date, L-type calcium channels and hERG appear to have been the most extensively studied channels with these techniques. In contrast, there are far fewer examples of computational models for the Na^+^ channel, although the recent publication about its atomic structure will change this gap shortly [12]. These three classes of ion channels have been studied as they represent not only useful targets with potential therapeutic applications, but also undesirable targets (“antitargets”) to be avoided because of the side effects they provoke when their function is altered.

This review discusses the methods that have been used for virtual ligand and target-based screening and profiling to predict biological activity. This includes the development of methods and databases, quantitative structure–activity relationships, pharmacophore searching, virtual screening, and molecular modeling. Furthermore, the review illustrates some of the applications of *in silico* methods directed to ion channels pharmacology. We also discuss the advantages and disadvantages of *in silico* methods with respect to *in vitro* and *in vivo* approaches for drug discovery and design. Our conclusion is that *in silico* pharmacology offers new and great opportunities for the discovery of new leads with predicted biological activity and improved therapeutic index.

## 2. Modeling Ion Channels

### 2.1. Target Availability and Selection

The receptor for *in silico* screening can be any macromolecule (protein or nucleic acid) whose spatial coordinates have been deposited in public databases such as RCSB and PDB. These databases are increasing daily, even for membrane proteins (http://blanco.biomol.uci.edu/mpstruc/listAll/list), including ion channels. There are actually more than 800 high resolution structures for membrane proteins (although only 300 are unique) and 60 of them correspond to ion channels. At present, several of these crystal structures such as the prokaryotic KcsA K^+^ channel transmembrane domain [13], full length KcsA in the closed [14] or open state [15], a chloride channel [16], cys-loop channels [17–19], the KvAP K^+^ channel [20], or the MthK channel [21], have provided opportunities for accurate modeling of homologue channels. The selection of such templates for modeling relies on the percentage of identity and similarity in the sequence alignment, the inserted and deleted regions, the atomic resolution of the template structure (3 Å or better) and, in general, the global resemblance of the target structure with the selected template. Cumulative evidence in homology modeling indicates that a sequence identity of 30% between the reference structure and the target channel is the limit for a reliable alignment. Below this limit, the protein model can have regions incorrectly folded in relation to the true structure [22]. This is of particular importance when a eukaryotic protein, in the absence of close homologues, is modeled with templates coming from prokaryotic organisms. In this sense, a critical step for improving the modeling of eukaryotic K^+^ channels was the recent availability of the eukaryotic potassium channel Kv1.2 structure at 2.4 Å in a lipid membrane-like environment [23], instead of the use of prokaryotic orthologs as templates.

### 2.2. Comparative Modeling

The availability of amino acid sequences of eukaryotic proteins and a myriad of bioinformatics tools available through the Internet make it possible to determine many protein features from their primary structure. For example, hydropathy plotting [24], transmembrane determination [25], secondary structure [26], disorder [27], glycosylation and phosphorylation prediction [28,29], and epitope scanning [30] are invaluable tools that can be applied in molecular modeling. The procedure of comparative modeling is performed by retrieving feasible structural templates and related sequences, and comparing them with the sequence of interest by pairwise or multiple sequence alignment [31–34]. Manual adjustment is often necessary to fit transmembrane domains and to move gaps to less conserved regions in proteins, *i.e.*, loops [2]. Generally speaking, homologue proteins used as templates for modeling targets are virtually identical in those areas where the homology is high. Conversely, loop regions exhibit a high degree of variability, even for closely related proteins [4]. Since most of the functional binding sites are located in loop regions, the modeling protocols have evolved to consider, for example, the orientation of known ligands during the homology modeling [35], thus improving the geometry of the binding sites. In addition, reliable models need to take into consideration protein and ligand flexibility.

Proteins and ligands have internal degrees of freedom and can adopt a myriad of conformational states. This is of outstanding interest since *in silico* procedures (*i.e.*, in VS) are expected to take into account the plasticity of the protein in the calculations [36], by using an ensemble of rigid receptor complexes [37] that can be later sampled by exhaustive conformational searches by molecular dynamics simulation [38], or by examining multiple binding-competent states of co-crystallized ligand-receptor complexes [39].

Another approximation for the limitation of the conformational space is the use of knowledge-based constraints, for example, the previous knowledge of conserved positions in protein-interacting peptides [40], the definition of a binding pocket based on a known structure [41], or the protocol for flexible peptide docking based on simulated annealing molecular dynamics approach [42]. Similarly, the incorporation of explicit water is highly significant. Whereas the implicit solvation models represent the solvent as a continuous medium to describe the average behavior of highly dynamics water molecules, several molecular mechanics applications, such as molecular dynamics and docking, use individual (explicit) water molecules in their calculations. The analysis of many crystal structures of protein complexes has revealed that in most cases a water molecule mediates contact between proteins and ligands, playing a role in ligand binding. In structure-based drug design, several attempts have been made to incorporate water in the calculations to improve the binding affinities in protein-ligand complexes, with impaired results [43,44]. In fact, the use of these explicit water molecules (in *i.e.*, molecular docking), is still challenging, since mediating waters can vary from ligand to ligand and waters found in the protein structure are displaced upon ligand binding [43]. In addition, molecular dynamics procedures on protein complexes including explicit water and entropic cost contribution have been applied [45].

**Figure 1.**
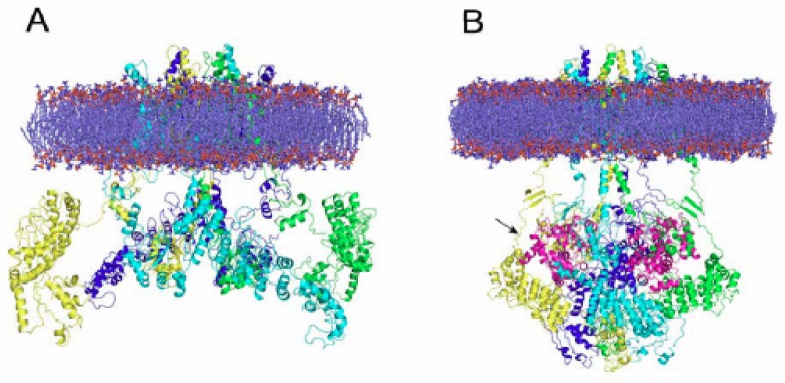
Tetrameric arrangement of the full human TRPV1 inserted into a model membrane. (A) Ribbon side view of the structural model of TRPV1 channel in the closed state. The extracellular loops, transmembrane and cytosolic regions (N- and C- terminus) are clearly depicted. In the closed state, the N-terminus does not interact with the C-terminus. (B) Side view of TRPV1 in the putative desensitized state. The flexibility of the N-terminal linker (black arrow) allows high mobility for the entire N-terminal to interact with the C-terminal via calmodulin (magenta) [55].

Comparative modeling has enabled the elucidation of 3D structures that would not actually be available otherwise. A case in point has been the modeling of channels activated by temperature, the so-called thermoTRP channels. TRPV1 and TRPM8, two members of the TRP family of ion channels [46–49] were recently fully modeled. TRPV1, a nonselective cation channel activated by heat (>42 °C), low pH and capsaicin, plays a key role in nociception, calcium homeostasis, hyperalgesia and neurogenic inflammation [50–54]. The molecular model of the full TRPV1 in the closed and desensitized states was published recently by assembling its major modules, namely the cytosolic N-, C-terminal domains and the membrane-spanning region. The model was validated using the extensive structure-function data available for TRPV1 [55]. In contrast to the resting state, where cytosolic domains were largely solvent-exposed, the desensitized state depicted a more interactive module, compatible with a Ca^2+^-calmodulin interaction with the cytosolic channel domains (see Figure 1).

Vistoli and his team [56] published the model of TRPM8, a channel activated by cold temperature, ligands such as menthol and icilin, positive membrane potential and the endogenous signaling phosphoinositides [57]. This channel, which is responsible for the sensations of innocuous cold [58–60], is also involved in normal noxious cold sensations and cold allodynia [61]. The model was used to explore the topology of N-terminal and their involvement in tetramerization, as well as the interrelation of adjacent monomers to unveil putative mechanisms of channel gating. Furthermore, both channels, TRPV1 and TRPM8 were used to explore their interactions capacities, as described in the following sections.

### 2.3. Finding the Active Sites

Several approaches to discover active sites, such as sequence-, template-, geometric- and energy-based, have been reported [62]. These methods exploit sequence and structural information either isolated or in combined form. For instance, the template-based methods identify binding sites by comparing patterns extracted from known binding sites. The localization of the binding site is important because it can be targeted by drug discovery. In geometric methods the determination of pocket size, shape and polarity with respect to a putative ligand is pivotal [63]. Several authors have suggested many parameters to identify such binding pockets, as pocket compactness, surface roughness and complexity, and total surface area [64]. The software available to determine putative active sites has been reported [62], *i.e.*, ICMPocketFinder [65] was used to explore binding pockets in modeling neurotransmitter transporters [66,67]. This strategy identified the determinants of rho1-GABA(C) receptor assembly by detecting the binding surfaces on the ligand-gated ion channel located in the transmembrane region. Interestingly, Tseng and Li developed an evolutionary approach to predict the binding site residues of proteins from primary sequences [68]. This is of particular interest for ion channels, since non-redundant protein databases have scarce structural information on these membrane proteins.

### 2.4. Candidate Ligands

Small commercially available ligands are listed in public databases, such as ZINC, that contains more than 14 million compounds [69] including modulators of ion channels. The question arising is whether or not these collections of molecules cover the entire chemical space, and which portions can be considered as drug-like molecules. Usually, molecules that meet the criteria for biological activity fulfill characteristics contained in the Lipinski's rule of five [70], or the more relaxed rules revised by Veber *et al.* [71]. The size of candidate ligands is important, as well as appropriate absorption, distribution, metabolism and excretion (ADME/Tox) properties. Thus, the collection of compounds can be filtered both by Lipinski's or Veber's rules, commercial availability, and by ADME/Tox properties. Compounds can be used as virtual libraries and synthesis can be postponed to a later stage for promising hits [4]. In addition, selected compounds should leave some room for optimization, matching the range of lead-like or fragment-like molecules [4,72]. Alternatively, the use of peptides as therapeutics (vaccine development, autoimmune diseases, neuroprotection, *etc.*) has been boosted by recent advances in peptide and peptidomimetic synthesis [73]. The fact that linear peptides (from highly disordered regions in proteins) mediate thousands of protein-protein interactions in signaling processes make the peptide discovery and development of high applicability and interest [74].

Recently it was demonstrated that peptides can be successfully used to target well-defined protein-protein interactions involved in TRPV1 channel function [75]. This protein has a cytosolic domain, referred to as the TRP region that is involved in subunit oligomerization and the functional coupling of stimulus sensing and gate opening. In that way, interfering with this protein interface could be used to modulate channel function. Indeed, it was demonstrated that palmitoylated peptides patterned after the membrane proximal TRP domain of TRPV1 behave as moderate and selective channel inhibitors and have been named as TRPducins. Furthermore, the use of cell-penetrating membrane peptides that target receptor domains proximal to the plasma membrane has been also used to modulate the activity of G protein-coupled receptors (GPCR) with high efficiency and selectivity [76]. Notably, some of these peptides, coined as pepducins, are leads for drug development.

## 3. Ligand-Based Methods. Quantitative Structure Activity Relationship (QSAR)

The analysis and recognition of QSAR has become an essential component of modern medical chemistry and pharmacology of ion channels, since very often the construction of reliable models is not feasible due to the lack of suitable templates. Basically, QSAR is an attempt to establish a correlation between a chemical structure and a biological effect of active biomolecules [2]. The representation of chemical structures is described through molecular descriptors [77] of different nature: 1-D descriptors encode generic properties as molecular weight, hydrophobic/hydrophilic partition coefficient, and molar refractivity, commonly related to a basic description of drug-like characters of the molecules [70,71]; 2-D descriptors (commonly referred as 2D-QSAR) predict physicochemical properties, and provide quantitative estimates of biological effects [78] from topological representations of biomolecules [79]; 3-D descriptors (3D-QSAR) derive directly from the 3-D structures of the molecules, depending on the conformation used and the flexible superposition of the molecules [80,81]. Three dimensional-QSAR offers a better representation of molecules interacting with proteins, and leads to statistically improved models. Several commercial tools are available to generate 3D-QSAR, such as Catalyst, CoMFA and CoMSIA. Unfortunately, the performance of QSAR approaches to relate the chemical property and the applicability within the chemical space is still far from the optimal results [82].

The pharmacophoric method is based on the analysis of a number of ligands known to act with a common mechanism of action. Ligand-based pharmacophore models are computed by extracting common features among three-dimensional structures of compounds which are known to interact with a target protein [83]. Other approaches use rule-based algorithms to recognize functional groups in the molecule under study and derive a list of putative changes the molecule can undergo based on rules extracted from databases [84]. Another computational task with high relevance is the prediction of effectors for members of protein families lacking any information about their ligands. The group of Jacoby developed the homology-based similarity searching, a methodology that predicts ligands starting from the target sequence and uses ligand information from target homologues [85].

Next we describe some examples of QSAR methods on ion channels involved in cardiovascular disorders, acute or chronic pain, epilepsy, migraine, and certain types of cancer. Mohan and coworkers studied a data set of compounds with N-type calcium channel blocking activity [86]. They used several descriptors (structure, ADME/Tox, thermodynamics, electrotopological) to derive a quantitative relationship between blocking activity and structural properties. They employed a genetic function to generate a 2D-QSAR model that, in turn, was trained on a set of 83 molecules and validated by a test set of 21 molecules. The resulting descriptors were used to reveal physico-chemical features of N-type calcium channels blocking activity.

The group of Nosko performed the evaluation of a set of hERG1 pore domain blockers by using 3D-QSAR in combination with receptor-based molecular docking approaches, and developed a pharmacophore model which provided a rapid assessment of the ability of compounds to block the channel [87]. The results were further validated by docking the hits against a homology model of hERG1 and *in silico* mutagenesis, in close agreement with the experimental data. Finally, the adenosine receptor, belonging to the G protein-coupled receptors (GPCR), served as a target for the identification and optimization of novel effectors using pharmacophoric models and 3D-QSAR ligand-based approaches [88].

## 4. Structure-Based Methods. Docking, Virtual Screening and Molecular Dynamics

### 4.1. Docking

The following methods require detailed structural knowledge of the receptor protein. The ability of docking methods to place ligands into a known native structure has been evaluated in recent papers [89–92], including ligand docking to proteins [93] and peptidic modulators [74]. In contrast with the basic rigid-body docking approach, a key factor for successful docking is the flexible ligand search approximation, where the degree of freedom of ligands, typically higher for peptides than for small molecules, is taken into account. Briefly, there are three categories of algorithms to treat ligand flexibility: (i) Systematic methods, which try to explore all the degrees of freedom in a molecule; (ii) Random or stochastic methods, which explore the conformational space by performing random changes in a ligand or a set of ligands, including three basic subtypes: Monte Carlo, Genetic Algorithm and Tabu Search methods; and (iii) Simulation methods, which employ the calculations of the solutions to Newton's equations of motions, including molecular dynamics and energy minimization methods [93]. The ability of docking methods to dock ligands into native structures has been recently reviewed [89,92]. While small molecule docking is achieved by several docking procedures, the docking of high flexible ligands (such as peptides) need further adaptation of the sampling strategy [94]. The limitation of the conformational space using constraints is often crucial for successful ligand docking. Constraints are usually derived from experiments such as NOE data or any other source of biological information (knowledge-based conserved positions, *etc.*).

Yuriev and coworkers recently described the homology modeling of nine G protein-coupled receptors, including dopamine, serotonin, histamine and muscarinic receptors, based on the structure of the β2-adrenergic receptor [95]. The ligand binding site was optimized with induced fit for side chain flexibility. The docking of a set of active antagonists showed an impaired ability of the models to improve the enrichment factors, which means that several models were correctly modeled while others required further refinement of the binding sites. Similarly, Pedretti *et al.* performed docking simulations to validate the model for the TRPM8 channel [56]. A small set of TRPM8 agonist and antagonist ligands were considered in docking simulations involving extensive rigid-body sampling. These studies resulted in a conformation where transmembrane S4 and S4-S5 linker play an important role in channel activation, in good agreement with mutational analysis. Using the same model for TRPM8 and molecular docking, it was confirmed the experimental observation that Y745 at the menthol binding site is critical for inhibition mediated by the antagonist SKF96365, but not for the antagonist BCTC, suggesting the existence of different binding sites for these ligands [58].

**Figure 2.**
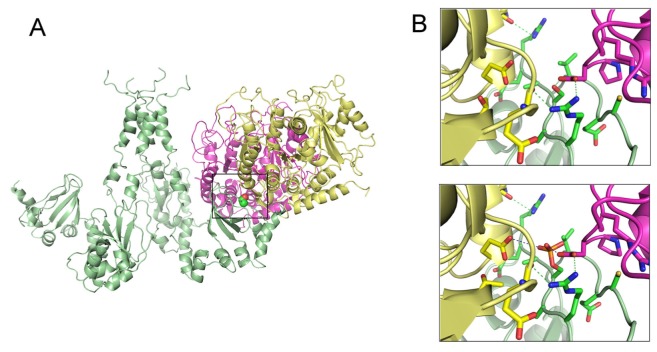
Protein-protein docking interaction of TRPV1 with tubulin. (A) Side view of the interaction of soluble α (yellow) and β (magenta) tubulin dimers with the tetrameric TRPV1 C-terminus (green). The PKCε phosphorylation site (Ser-800) is indicated by the black box. (B) Enlarged view of the tubulin-TRPV1 interaction at Ser-800 in the non-phosphorylated (upper) and phosphorylated state (lower). The interaction is stabilized among others by side chain and main chain hydrogen bonds (dotted green lines). In contrast, phosphorylation of Ser-800 by PKCε clashes sterically (dotted blue lines) with several side chains, avoiding interaction [96].

Studies of protein-protein docking prediction had been used to describe the role of TRPV1 in the estrogen signaling to induce protein kinase C-ε (PKC-ε) activity, a central component in various models of pain sensitization [96]. The interaction of TRPV1 with the cytoskeleton is hindered by protein phosphorylation at S800, suggesting that the C-terminus of TRPV1 is a signaling intermediate of estrogen and PKC-ε. This interaction plays an important role in microtubule-dependent pain sensitization, as well as in microtubule stability (Figure 2).

Docking studies were also used to describe the pharmacological activity of both novel and traditional compounds on acetylcholine receptor, a member of Cys-loop receptor superfamily of pentameric ligand-gated ion channels. Arias *et al.* confirmed the site of action of novel amide derivatives on human α7 AChR as the interface between α7 subunits, 12 Å away from the agonist locus and with potential therapeutic relevance [97]. The anesthetic binding sites of bacterial nicotinic acetylcholine receptors have been exploited by Chen *et al.* using an experimental and computational hybrid approach [98]. Docking analysis revealed several binding sites for halothane and thiopental (fluorescence quenchers) at extracellular and transmembrane domains, as well as the extracellular-transmembrane interface. In addition, the study revealed intrasubunit sites for halothane and intersubunit sites for thiopental binding, showing that anesthetic binding disrupts critical interactions for channel gating and destabilizes the open channel conformation.

Several lines of work deal with the interaction of drugs with voltage-gated ion channels. Noujaim *et al.* studied the voltage-gated inward rectifier ion channel Kir2.1 by molecular modeling and ligand docking to describe the structural basis of action of anti-fibrillatory compounds chloroquine and quinidine [99]. The work showed an effective block of chloroquine by binding at the center of the ion permeating vestibule, while quinidine only blocked the ion movement partially, in good agreement with experimental results. The human ERG potassium channel, which has a major pharmaceutical relevance, was homology modeled to conduct molecular docking experiments in order to describe the structural basis of binding of several blockers to the channel [100]. The study showed two main interaction regions that can be further used for experimental test and/or specific drug design. KcsA channel docking studies were performed to simulate the inactivating *Shaker* B ball peptide in complex with an open pore KcsA. The ball peptide was modeled as a β-hairpin, as suggested by FTIR experiments and the open KcsA was modeled with the MthK channel. The simulation correctly predicted the molecular interaction map obtained experimentally with saturation transfer difference NMR methods [101]. Finally, docking approaches with amiloride directed to ASIC1 were used to discover potential interaction sites within the protein and predict analogs of amiloride with different affinities [102]. Although the results are valid for additional computer predictions, such as virtual screening with chemical libraries, this and other theoretical studies should be experimentally verified to confirm the validity of the binding site prediction.

### 4.2. Virtual Ligand Screening (VS)

Virtual screening is a process conceived to score a long list of molecules with putative affinity for a target [103]. It was developed as an alternative to HTS, and has become an important part of the lead discovery process [104]. It requires structural information on the targets and/or on bioactive ligands, and the different types of virtual screening methods depend on the structural information available [105]. Following the principle that similar molecules exhibit similar properties [106], all molecules in a database can be scored in relation to a real bioactive ligand and ranked to offer a non-random list of hits for experimental testing. The most commonly used approaches in virtual screening are the flexible superposition of molecules onto a reference bioactive molecule [107] and pharmacophore similarity [83], defined as the 3D arrangement of molecular features needed for bioactivity [108]. Pharmacophore approaches have been extensively employed and are considered of high versatility to be used with complex biological targets such as ion channels [109]. When the target structure is available by means of crystallography or derived by homology modeling, the virtual screening provides a nice picture of the interaction at a molecular level. However, there are still two main challenges to overcome: a) the determination of the correct conformation and ligand orientation (docking), and b) the estimation of the binding affinity (scoring) [109]. Docking methods benefit from the fact that there is a growing increase of protein-ligand complexes determined experimentally, whose conformational information is incorporated to guide the docking of ligand to binding sites [110]. A large list of resources to assist structure-based virtual screening was reported [111].

An ion channel targeted by virtual screening approaches is the nicotinic acetylcholine receptor α7, a potential therapeutic target for Alzheimer's disease and other neurological disorders. Recently, this channel was modeled by Dey and Chen [112]. The authors performed a high throughput virtual screening by flexible ligand docking of a large set of compounds in three different binding sites of α7 nicotinic receptor. The experimentally verified modulators ranked correctly in the computational approach, pointing to virtual screening as a reliable tool to infer new modulators.

NMDA (*N*-methyl-d-aspartate) receptors are considered attractive targets for Alzheimer's disease, schizophrenia, epilepsy and chronic pain. Krueger *et al.* validated different techniques for virtual screening to identify potential competitive antagonists of the NMDA receptor glycine binding site located in the NR1 subunit [113]. Molecular docking, pharmacophore search, pharmacophore QSAR and Bayesian machine learning were used for virtual screening of 4.6 million chemicals. The resulting hits were experimentally tested and novel active compounds were identified. Mony *et al.* identified a novel NMDA receptor antagonist selective for heteromeric NR1-NR2B receptors using a virtual high-throughput screening approach based on a quantitative pharmacophore model of known antagonists [114]. Interestingly, since the first compound developed to avoid NMDA receptor overactivation failed in clinical trial due to strong side effects, low oral bioavailability or poor PK profile, the finding of alternative antagonists is of outstanding interest. These authors identified a novel compound with an original central core that inhibited selectively the NR2B subunit, with improved ADME/Tox parameters. Finally, potassium channels are attractive targets for drug discovery because of its implication in essential processes in biological systems. Pegoraro *et al.* [115] identified blockers of Kv1.3 potassium channels using a KcsA template for homology modeling, and a virtual screening approach. The new compounds displayed inhibitory effects on T-cell and keratinocytes proliferation and immunosuppressant activity. Liu *et al.* [116] constructed a eukaryotic model of voltage-gated potassium channel based on KcsA template to search for new blockers of potassium channels from large databases of compounds. Twenty hit candidates were experimentally validated. Six compounds were potent blockers of delayed rectifier and fast transient potassium currents when applied externally in a whole-cell voltage-clamp recording system, indicating that VS is an invaluable tool to discover novel and structurally diverse compounds targeted to the outer mouth of these channels.

### 4.3. Molecular Dynamics (MD)

Molecular dynamics has become the most popular theoretical method for ion channel studies because it can provide an atomic picture directed to rationalize the mechanisms of ion conduction and specificity [117]. The different states of ion channels and the gating processes can be studied in high spatial and temporal resolution, as reviewed in [7]. The advantages of MD studies on ion channels include also the understanding of ion permeation control induced by external factors, such as voltage, pH concentration gradients and drugs, as well as the prediction and analysis of variations in the channel behavior after mutagenesis, allowing a better understanding of genetic disorders that cause channelopathies (impaired ion channel function) and the application of drug discovery principles.

Probably, the most studied channel using MD methods is the prokaryotic potassium channel KcsA, in an attempt to determine the ion coordination numbers and ion specificity. These studies started with free energy perturbation calculations on the selectivity filter of KcsA [118] to show an intrinsic selectivity for K^+^ versus Na^+^. Although the channel selectivity can be explained by equilibrium thermodynamics (comparing the free energy of ions in solution or in the protein environment), the nature of the selectivity is still elusive [117]. Ion selectivity was first explained based on geometrical constraints imposed by the selectivity filter to accommodate ions [13]. However, selectivity studies using MD simulations demonstrated that the selectivity filter is quite flexible to accommodate K^+^ or Na^+^ [119], being the protein able to sense the ion size. In addition, it was postulated that the repulsion between carbonyl groups in the filter plays a key role in ion selectivity [120], being higher for Na^+^ than for K^+^; and that these carbonyl groups favor higher coordination numbers for ions in water solution [121,122]. A recent study using QM/MM simulations, however, found that coordination numbers for K^+^ and Na^+^ are quite similar in solution or in the selectivity filter, indicating that the ion coordination variation between water and filter is not a requirement for selectivity [123]. Cordero-Morales *et al.* used a hybrid experimental and simulation approach to characterize the inactivated state of KcsA [124]. This study concluded that the conductive conformation of the filter is unstable and identified an inactivated state of low free energy in which a binding site for potassium is disrupted. Similarly, non-conductive conformations have been also identified in simulations in other potassium channels, as KirBac1.1 [125]. Simulations of the voltage-sensor domain of Kv channels have shown that positively charged residues are solvated inside the membrane [126], as well as the existence of important deformations of the lipid bilayer around the protein [127] that may be essential for stimulus sensing.

Mechanosensitive channels are involved in many biological processes, such as hearing, touch sensation, and cell-volume regulation. The availability of prokaryotic structures facilitates the modeling and MD simulations in this protein family. The study of MscL protein using a continuum mechanics model, described interactions of neighbor MscL proteins to regulate the clustering and the open probability of the channel [128]. Other mechanosensitive channel, McsS, was simulated to assess the transition from closed to open conformation with explicit transmembrane potential [129], suggesting different opening steps to reach fully channel conductance, a suggestion that remains to be tested experimentally.

Molecular dynamics of electron microscopy structures of nicotinic AcChR channels showed that the transmembrane domains contain internal binding sites for regulators of membrane fluidity such as cholesterol, and that the binding of the sterol to these sites increases the stability of the channel structure [130]. Other studies on ion selectivity in AcChRs attributed the interaction of ions with charged residues located in the inner and outer mouths of the pore, favoring or disfavoring electrostatically the pass of ions [131]. Studies on acid-sensing ions channels at different ionic concentrations were used to identify ion binding sites, as well as changes in protein conformation after ion binding [132]. A tetrodotoxin-based P-loop domain model of a sodium channel evaluated with Monte Carlo minimization was used to study the role of P-loop in the slow inactivation [133]. The modeling in the presence of sodium predicted ions interacting with outer negatively-charged rings and ions transiting to the selectivity filter ring. Additionally, the model in the absence of sodium correctly predicted the disruption of the permeable configuration. The human α1 glycine receptor studied by Cheng *et al.* [134] using the refined structures of nicotinic AcChR revealed different states of the channel and suggested that channel closure may be conducted by rotation or tangential tilting of the transmembrane helix 2 (TM2). The potentiating effect of ethanol on α1 glycine receptors was also studied [135,136]. The authors were able to observe spontaneous binding of ethanol between and within receptor subunits, as well as the equilibrium ligand exchange, by performing two microsecond-scale simulations. The studies suggested that ethanol stabilized the glycine receptor model on a putative open form of the ligand-gated channel, explaining the effects of allosteric ligand binding on these receptors.

## 5. Remarks

The understanding of the cellular machinery for communication and regulation, and how gene products interact at atomic level are a major goal in the post genomic era. Computational models applied to important biological targets have contributed considerably to our knowledge of phenomena involved in biological processes. The computational study of ion channels has enhanced our understanding of the mechanism underlying ion transport across the membrane, ion selectivity, fast channel inactivation and lipid interactions, as well as protein conformation and dynamics. Accordingly, modeling and theoretical calculations offer a powerful complementary approach to support *in vitro* experimental studies.

**Figure 3.**
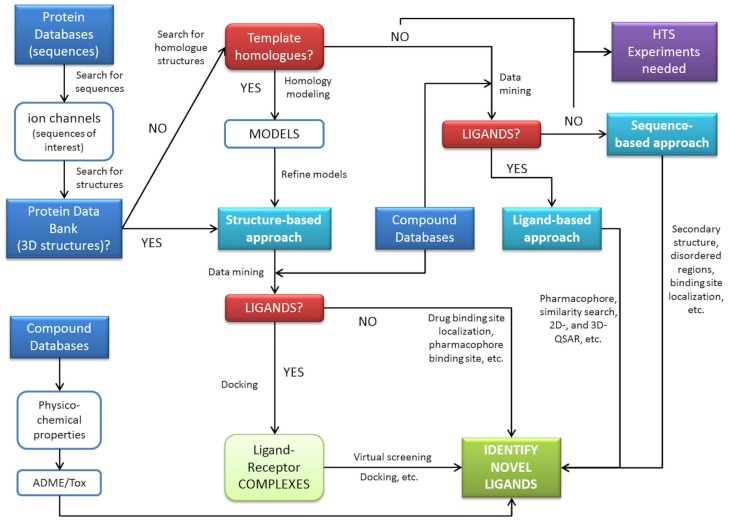
A schematic view of general procedures for *in silico* pharmacology. Three main procedures, structure-, ligand-, and sequence-based approaches are interconnected with databases for a general goal, the discovery of novel compounds to modulate proteins.

The examples provided in this review demonstrate the ability of *in silico* methods to obtain extra information from available data. These methods have the capacity to be used to search large databases and suggest molecules for rapid testing, and often the selected hits represent a significant enrichment over a random selection of molecules. A general scheme for *in silico* prediction of interactions can be derived by connecting steps and procedures commonly used in biocomputation (Figure 3). This demonstrates some of the key roles of computational technologies that can assist pharmacology of ion channels. These roles include finding new antagonists or agonists for a target using an array of methods either in the absence or presence of an available structure for the target. Computational methods may also aid in understanding the underlying biology using network/pathways based on annotated data (signaling cascades), the drug-target network connectivity to understand selectivity, the integration with other models for PK/PD and finally, the emergence of systems biology.

These methods are gaining acceptance in the pharmaceutical industry as a cost-effective and timely strategy for analyzing large sets of chemicals. In general, the advantages of *in silico* methods are: (i) a pronounced reduction in the number of molecules tested through database searching to find inhibitors or substrates; (ii) an increased speed of reliable prediction of most pharmaceutical properties from the structure of the molecule alone; (iii) a greater capacity to suggest bench experiments; (iv) a reduction in animal experimentation, and (v) important savings in research costs.

On the other hand, the main shortcoming of *in silico* pharmacology is the availability of ligands and targets 3D structures. Even with the accessibility of crystal structures for mammalian ion channels, there are still some caveats including protein flexibility, molecule conformation and promiscuity, which compromise the reliability of the predictions from the structural models.

Despite the above limitations, the future of *in silico* pharmacology looks bright and it will be pivotal to advance drug discovery programs in the ion channel field. Nonetheless, in addition to improving the techniques for solving ion channels structures, there is a need for developing better software since the modeling of channels as drug targets rely on both structural templates and software [134]. The bioinformatics tools for modeling receptor targets, molecular dynamics between the drug and the target and, in general, the wealth of software with biological applications will benefit from more powerful computer hardware. However, it is mandatory the need to calibrate, characterize and standardize the software to produce a better integration between experimental and computational approaches in structural biology [137].
